# Complete mitochondrial genome of *Parupeneus barberinus* (Perciformes, Mullidae): mitogenome characterization and phylogenetic analysis

**DOI:** 10.1080/23802359.2017.1398613

**Published:** 2017-11-07

**Authors:** Ha Yeun Song, Young Se Hyun, Seonmi Jo, Seung Hyun Jung, Hyun-Ju Hwang, Seong-Yong Kim, Hye Suck An

**Affiliations:** a Department of Genetic Resources Research, National Marine Biodiversity Institute of Korea, Janghang-eup, Republic of Korea;; b Department of Taxonomy and Systematics, National Marine Biodiversity Institute of Korea, Janghang-eup, Republic of Korea

**Keywords:** Mitochondrial genome, Perciformes, Mullidae, *Parupeneus barberinus*

## Abstract

*Parupeneus barberinus* is a tropical/subtropical reef-dwelling marine fish belonging to the family Mullidae. Herein, we report the first sequencing and assembly of the complete mitochondrial genome of *P. barberinus*. The complete mitochondrial genome is 16,560 bp long and has the typical vertebrate mitochondrial gene arrangement, consisting of 13 protein-coding genes, 22 tRNA genes, two rRNA genes, and a control region. Phylogenetic analysis using mitochondrial genomes of 18 species showed that *P. barberinus* is clustered with *P. multifasciatus* and *P. chrysopleuron* and rooted with other Mullidae species. This mitochondrial genome provides potentially important resources for addressing taxonomic issues and studying molecular evolution.

The dash-and-dot goatfish, *Parupeneus barberinus* (Perciformes: Mullidae), is widely distributed throughout the Indian and western Pacific Oceans, from the east coast of Africa to Australia and Tuamotus (Randall 2004). It is one of the most abundant species of *Parupeneus* belonging to the family Mullidae (Myers 1999). Although previous molecular phylogenetic analyses indicated that the Mullidae (goatfishes) formed a monophyletic group together with Dactylopteridae (flying gurnards), Syngnathoidei (pipefishes and seahorses), and Aulostomoidei (trumpetfishes and cornetfishes) (Betancur-R et al. 2013; Near et al. 2013; Song et al. 2014), the phylogenetic position of Mullidae within Percomorpha remains controversial. To the best of our knowledge, this is the first study to determine the complete mitochondrial genome of *P. barberinus*, and to analyze the phylogenetic relationship of this species with members of Syngnathiformes.

The *P. barberinus* specimen used in this study was collected from Chuuk ST 1, Micronesia (7.27N, 151.54E). Total genomic DNA was extracted from tissue of the specimen, which has been deposited in the National Marine Biodiversity Institute of Korea (Voucher no. MABIK 0000613). The mitogenome was sequenced and assembled using Illumina Hiseq 4000 sequencing platform (Illumina Inc., San Diego, CA) and *SOAP denovo* assembler at Macrogen Inc. (Seoul, Korea), respectively. The complete mitochondrial genome was annotated using MacClade ver. 4.08 (Maddison and Maddison 2005) and DNASIS ver 3.2 (Hitachi Software Engineering, Tokyo, Japan).

The complete mitochondrial genome of *P. barberinus* (GenBank accession no. AP018401) is 16,560 bp long, and includes 13 protein-coding genes, 22 tRNA genes, two rRNA genes, and a control region. The *ND6* gene and eight tRNA genes are encoded on the light strand. The overall base composition of the heavy strand is 26.62% A, 30.33% C, 18.26% G, and 24.79% T. Similar to the mitogenomes of other vertebrates, the AT content is higher than the GC content (Saccone et al. 1999). All tRNA genes can fold into a typical cloverleaf structure, with lengths ranging from 61 to 74 bp. The 12S rRNA (953 bp) and 16S rRNA genes (1693 bp) are located between tRNA^Phe^ and tRNA^Val^ and between tRNA^Val^ and tRNA^Leu(UUR)^, respectively. Of the 13 protein-coding genes, 11 begin with an ATG start codon; the exception being the *ND2* and *COI* gene, which start with GTG. The stop codon of the protein-coding genes is TAA in *COI*, *ND4L,* and *ND5*; TAG in *NDI*, *ATP8,* and *ND6*; TA in *ND2*, *ATP6*, *COIII,* and *ND3*; and T in the remaining three genes. The control region (882 bp) is located between tRNA^Pro^ and tRNA^Phe^.

Phylogenetic trees were constructed by the maximum-likelihood method using MEGA 7.0 software (Kumar et al. 2016) for the newly sequenced genome and a further 17 complete mitochondrial genome sequences downloaded from the National Center for Biotechnology Information. We confirmed that *P. barberinus* is clustered with *P. multifasciatus* and *P. chrysopleuron*, and formed monophyletic group with other two syngnathoid families (Fistulariidae and Dactylopteridae) with high statistical support (Figure 1). This mitochondrial genome provides potentially important resources for addressing taxonomic issues and studying molecular evolution.

**Figure 1.**
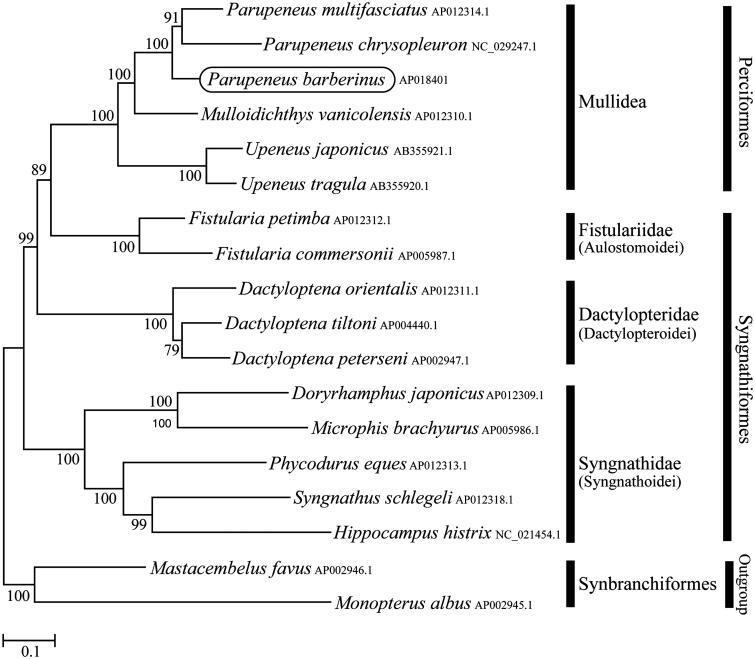
Phylogenetic position of *Parupeneus barberinus* based on a comparison with the complete mitochondrial genome sequences of 17 species. The analysis was performed using MEGA 7.0 software. The accession number for each species is indicated after the scientific name.
